# The influence of metabolic imbalances and oxidative stress on the outcome of critically ill polytrauma patients: a review

**DOI:** 10.1186/s41038-017-0073-0

**Published:** 2017-03-07

**Authors:** Alexandru Florin Rogobete, Dorel Sandesc, Marius Papurica, Emil Robert Stoicescu, Sonia Elena Popovici, Lavinia Melania Bratu, Corina Vernic, Adriana Mariana Sas, Adrian Tudor Stan, Ovidiu Horea Bedreag

**Affiliations:** 1 Faculty of Medicine, Victor Babes University of Medicine and Pharmacy, Str. Eftimie Murgu Nr. 2, Timisoara, 300041 Timis Romania; 2 Clinic of Anaesthesia and Intensive Care, Emergency County Hospital “Pius Brinzeu”, Bd. Liviu Rebreanu Nr.156, Timisoara, 300736 Timis Romania; 3 Faculty of Pharmacy, Victor Babes University of Medicine and Pharmacy, Str. Eftimie Murgu Nr. 2, Timisoara, 300041 Timis Romania; 4 Faculty of Dental Medicine, Victor Babes University of Medicine and Pharmacy, Str. Eftimie Murgu Nr. 2, Timisoara, 300041 Timis Romania

**Keywords:** Critically ill, Energy expenditure, Indirect calorimetry, Metabolic disaster, Oxidative stress, Overfeeding, Underfeeding, Polytrauma

## Abstract

The critically ill polytrauma patient presents with a series of associated pathophysiologies secondary to the traumatic injuries. The most important include systemic inflammatory response syndrome (SIRS), sepsis, oxidative stress (OS), metabolic disorders, and finally multiple organ dysfunction syndrome (MODS) and death. The poor outcome of these patients is related to the association of the aforementioned pathologies. The nutrition of the critically ill polytrauma patient is a distinct challenge because of the rapid changes in terms of energetic needs associated with hypermetabolism, sepsis, SIRS, and OS. Moreover, it has been proven that inadequate nutrition can prolong the time spent on a mechanical ventilator and the length of stay in an intensive care unit (ICU). A series of mathematical equations can predict the energy expenditure (EE), but they have disadvantages, such as the fact that they cannot predict the EE accurately in the case of patients with hypermetabolism. Indirect calorimetry (IC) is another method used for evaluating and monitoring the energy status of critically ill patients. In this update paper, we present a series of pathophysiological aspects associated with the metabolic disaster affecting the critically ill polytrauma patient. Furthermore, we present different non-invasive monitoring methods that could help the intensive care physician in the adequate management of this type of patient.

## Background

Critically ill polytrauma patients show a series of associated pathophysiologies that can increase both morbidity and mortality [1, 2]. The most frequent and best researched of these pathologies are systemic inflammatory response syndrome (SIRS), acute respiratory distress syndrome (ARDS), sepsis, and multiple organ dysfunction syndrome (MODS) [3–6]. Moreover, secondary to these pathologies, these patients quickly develop oxidative stress (OS) and severe metabolic disorders characterized in particular by hypermetabolism [7, 8]. Recent studies have shown a strong link between nutritional imbalance and the outcome of critical patients. Huang et al. reported that over 90% of critical patients are malnourished 14 days after admission to the intensive care unit (ICU) [9].

From a metabolic perspective, a critically ill polytrauma patient is characterized by an increase in energy expenditure (EE), an increase in insulin resistance, and a decrease in protein synthesis. By drawing together the pathophysiological aspects of primary and secondary traumatic injuries, clinical aspects of muscle weakness, increased immobility, hard recovery, and increased ventilation time are described [10–13].

To optimize nutritional therapy according to the needs of each patient, a number of monitoring methods have been developed. One of the best known is indirect calorimetry (IC), based mainly on monitoring oxygen consumption (VO_2_) and carbon dioxide production (VCO_2_) [10, 14].

In this updated paper, we present an overview of the pathologies associated with the metabolic disorders developed in critically ill polytrauma patients. We also present several methods that are useful in the monitoring of the nutritional balance of these patients, specifically of EE.

## Review

### Pathophysiological and genetic-related aspects

**Fig. 1:**
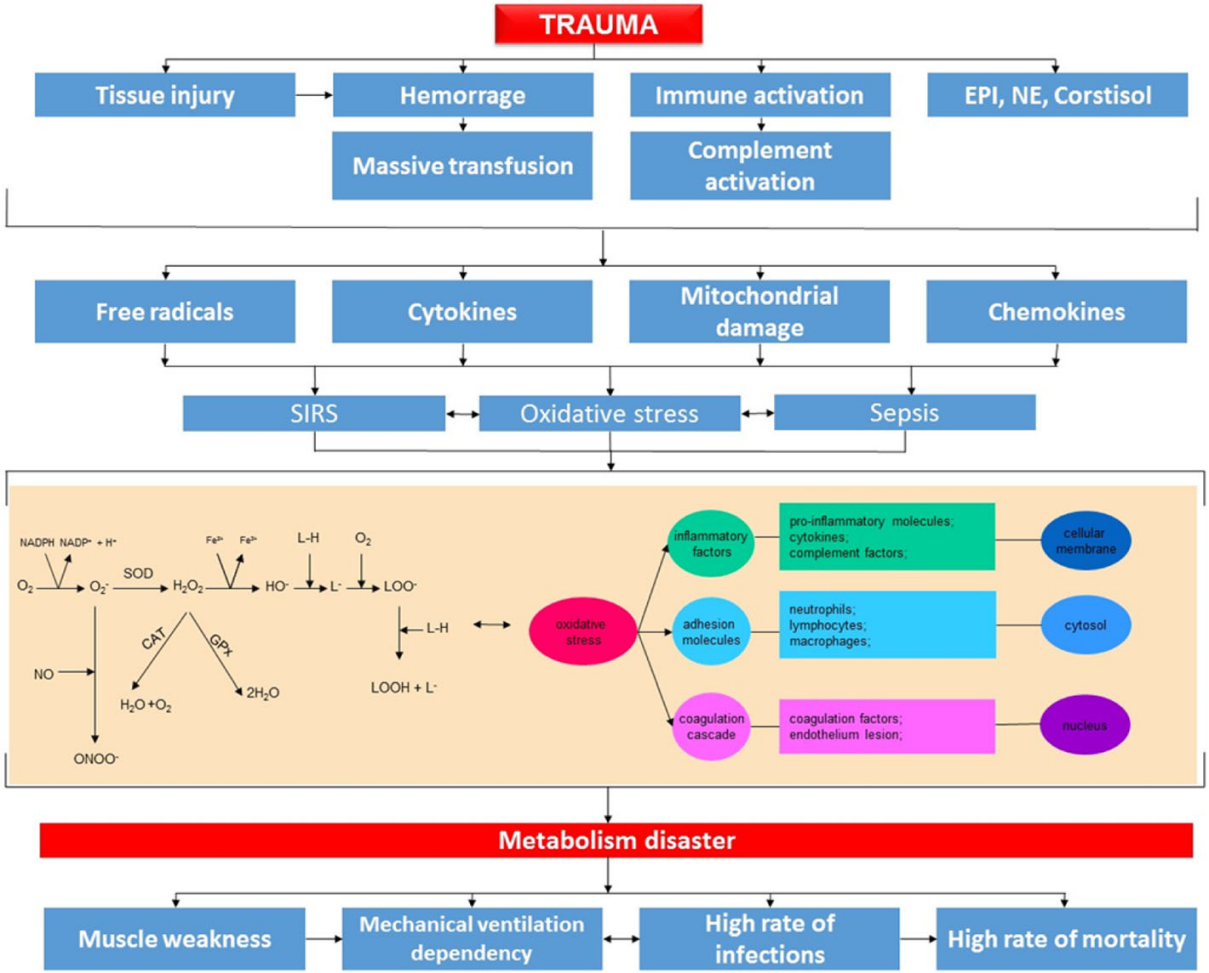
Pathophysiologies associated with trauma and their influence on metabolic disaster. Pathophysiological links between trauma, proinflammatory status, pro-oxidative status (oxidative stress), and clinical outcomes. *EPI* epinephrine, *NE* norepinephrine, *SIRS* systemic inflammatory response syndrome

Critically ill polytrauma patients present with several complex disorders that lead to a significant decrease in survival rate (Fig. 1).

In terms of metabolic status, in the event of inappropriate nutritional therapy, critically ill patients will suffer from either malnutrition or overfeeding. Smallwood et al. conducted a study on the determination of the resting energy expenditure (REE) of critically ill pediatric patients by IC using E-COVX gas exchange modules (GE Healthcare, Waukesha, WI). The Weir equation was used for REE calculation (REE = [VO_2_ × 3.941 + VCO_2_ × 1.106] × 1440). The energy supply for 63% of patients was under the measured EE, 16% met the measured EE requirement, and 21% were overfed above the measured EE [15].

Tavladaki et al. conducted a study regarding the evaluation and monitoring of metabolic imbalances using cellular biomarkers, such as the expression of ATP, NO_2_^−^, and continuous monitoring for EE, using the gas module E-COVX (GE Healthcare, Helsinki, Finland) in critical patients with sepsis and post-traumatic SIRS. Following that study, it was shown that patients with sepsis and SIRS present with a predominantly hypometabolic profile. Moreover, patients with sepsis presented lower production of ATP than healthy controls (184 ± 133 vs. 895 ± 863 nM) [16].

Oxidative stress (OS) is another pathology found in a high percentage of critically ill patients. Numerous studies have highlighted the impact of OS on the outcome of these patients [17, 18]. They have reported numerous OS-associated pathologies that are responsible for increased morbidity and mortality rates in critically ill polytrauma patients. Several studies have shown that increased OS is associated with a major metabolic imbalance [19].

From a biochemical perspective, the most important free radicals are the reactive oxygen species (ROS), the reactive lipid species (RLS), and the reactive nitrogen species (RNS). Under physiological conditions, the human body is protected from OS by the endogenous enzymatic system, represented by glutathiones (GSHs), catalases (CATs), glutaredoxins (Grxs), thioredoxins (Trxs), and peroxiredoxins (Prxs) [20–22]. Together with the augmentation of the redox status, a series of biological cellular systems are affected, such as DNA and protein destruction or the lipid status, through their destruction and the implicit production of new free radical species. Regarding the evaluation and monitoring of OS using specific serum biomarkers, the most representative to date are malondialdehyde (MDA) and 4-hydroxynonenal (4-HNE) for lipid peroxidation, while for protein peroxidation, the most important ones are carbonyl groups and nitrotyrosine [23–26]. Other biomarkers that could offer answers in regard to pro-oxidative and proinflammatory status are specific cytokines such as interleukin 1 (IL-1), interleukin 2 (IL-2), interleukin 6 (IL-6), interleukin 8 (IL-8), and tumor necrosis factor alpha (TNF-α). Furthermore, specific biomarkers for OS, such as glutathione disulfide (GSSG) and reduced glutathione (GSSH), have also been reported [27–29]. However, at present, there are a number of studies that describe other methods for evaluating OS. Bjugstad et al. studied the oxidation-reduction potential (ORP) in patients with traumatic brain injury. They measured the static ORP (sORP) to reflect oxidative stress and the induced capacity ORP (icORP) to reflect induced oxidative stress. In that study, they showed that icORP at day 4 is useful for determining the clinical outcome (*p* < 0.05). Analyses were performed using the RedoxSYS system to measure the ability to transfer electrons from an antioxidant to an oxidant [30].

Critically ill polytrauma patients present an increased proinflammatory status, leading to an increased incidence of multiple organ dysfunction syndrome (MODS) development in such cases [5]. Under these conditions, a number of metabolic disorders and biochemical mechanisms are severely affected due to the aggressive reactions induced by free radicals (FRs) and other cellular proinflammatory mediators. An important protection factor for the cell is glutamine. In accentuated pro-oxidative and proinflammatory conditions, the serum levels of glutamine decrease, leading to the need for exogenous administration. Grintescu et al. studied the effects induced by the administration of specific nutrients in critically ill patients experiencing hyperglycemia. After the study, they highlighted that glutamine supplementation is associated with a significant decrease in the incidence of hyperglycemia episodes. They also reported a significant decrease in the daily insulin dose required for the stabilization of blood glucose levels [31]. In the case of a critically ill patient, the metabolic balance is maintained through complex mechanisms of energy substrate use. Pathophysiological changes are significantly reflected in the metabolic balance and therefore take their toll on the clinical aspects of these patients. From a clinical perspective, we can identify a number of changes in terms of drastic increases in EE and in glucose concentration and a decrease in muscle mass. The inflammatory response is also involved in modifying the metabolism in these patients. Albacker et al. conducted a similar study, which showed a decrease in IL-6, IL-8, and TNF-α concentrations in patients who had insulin administered to modulate the metabolic balance [32]. Jeschke et al. conducted a similar study in patients with severe burns and identified a normalization of proinflammatory factors by modulating the metabolism through insulin therapy [33].

Table 1 summarizes the clinical consequences of inadequate nutrition in critically ill patients.

**Table 1:** Clinical consequences due to severe metabolic imbalance

Metabolic imbalance	Observations	References
Underfeeding	Negative energy balance associated with poor outcome of critically ill patients	[44]
	Nutrition intolerance is associated with a high mortality rate	
	Hitting a specific caloric target for each patient is associated with improved outcomes and lower mortality rates	
Overfeeding	Overfeeding is associated with hypercapnia	[59, 60]
	Excess nutrient administration is associated with lower survival rate	
	A longer time on mechanical ventilation and in the ICU is reported	
	Hyperglycemia and hypertriglyceridemia were highlighted	
	A high number of cases of metabolic acidosis and hypertonic dehydration associated with overfeeding have been reported	
Autophagy	Associated with insufficient degradation of protein structures and malfunctioned mitochondria	[61]
	Accelerated muscle destruction	
	Difficult biochemical and pathophysiological systems recovery in case of patients with MODS	
	The immune system is affected and sepsis is accelerated	
	Affection of the endogen antioxidant system with augmented pro-oxidative and proinflammatory status	
Re-feeding	A severe change in electrolyte balance is reported	[62, 63]
	Fluid and sodium ions retention are associated with heart failure and respiratory failure	

A high number of genetic factors are involved in the metabolic balance. Thus, a series of biochemical and genetic systems responsible for the metabolic mechanisms of cholesterol, glucose, and insulin were identified. Among them, the most common are peroxisome proliferator-activated receptors (PPARs), sterol-regulatory element-binding proteins (SREBPs), liver X receptors (LXRs), and forkhead box protein O1 (FOXO1) [34]. Recent studies have shown a strong relationship between the expression of miRNAs and the metabolic activity of critically ill patients. One of the most intensely researched species involved in lipid metabolism was miRNA-122 [35]. Other species involved in lipid metabolism include miRNA-33a and miRNA-33b [36]. Regarding the insulin metabolism, Baroukh et al. showed increased expression levels for miRNA-375 [37]. Another species involved in the biochemical pathways of insulin is miRNA-124a [37], in a strong relationship with miRNA-124a activity. Lu et al. showed elevated expression levels of miRNA-223, which is involved in glucose metabolism through SLC2A4 [38]. Also in relation to glucose, studies have revealed important implications of miRNA-29 [39]. Regarding OS production in critically ill patients, numerous studies report significant implications for the outcome of these patients. Bedreag et al., in a study of the influence of OS on the outcomes of critically ill polytrauma patients, reported strong and statistically significant correlations between OS-blocking through antioxidant therapy and the decreased expression of specific biochemical markers, shortened ICU days, and decreased mortality [40]. Sepsis is a leading cause of death in the ICU [41, 42], due mainly to the associated pathophysiologies that these patients develop, which will ultimately lead to the development of MODS and finally to death. In septic patients, the metabolic status is significantly altered and the EE is completely changed. Feferbaum et al. conducted a study on REE in neonatal patients with sepsis. They reported a significant increase in EE from 49.4 ± 13.1 kcal/kg/day to 68.3 ± 10.9 kcal/kg/day in the acute phase of sepsis. Moreover, during the acute phase of sepsis, they revealed a marked increase in VO_2_ and correspondingly VCO_2_. Thus, they highlighted statistically significant differences (*p* < 0.01) for VO_2_ that increased from 7.4 ± 1.9 ml/kg/min to 10 ± 1.5 ml/kg/min. The case of VCO_2_ was similar, showing a significant increase from 5 ± 1.7 ml/kg/min to 7.4 ± 1.5 ml/kg/min [43]. Alberda et al. conducted a study on the relationship between the amount of administered protein and the clinical outcomes of critically ill patients. This trial included 2772 mechanically ventilated patients, who were then administered 1034 kcal/day and 47 g protein. The study reported a significant decrease in the time spent on a mechanical ventilator and a significant decrease in mortality at 60 days [44].

Villet et al. conducted a similar study on the links between underfed critically ill patients and their outcome. In this study, EE monitoring was performed using electronic equipment based on indirect calorimetry. The results showed a strong correlation between mortality rates and the energy deficit of these patients [45]. Allingstrup et al. conducted a similar study on correlations between the nutrient doses administered and the outcomes of critically ill patients. Metabolic status monitoring was performed using a Deltatrac II (MBM-200, Datex-Ohmeda, Helsinki, Finland) and a CCM Express Indirect Calorimeter (MedGraphics, St. Paul, MN, USA). The study showed a significant decrease in mortality in patients who were administered a higher dose of proteins and amino acids [46].

### Energy demand evaluation and monitoring methods

One of the most widely used methods for calculating energy demand in critically ill pediatric patients is represented by Schofield’s predictive equations, based on a linear equation that includes variables such as the weight, height, and sex of the patient. It has been shown that in such patients, these predictive equations can have large errors due to the complexity of the associated pathophysiologies. van der Kuip et al. conducted a study on the EE monitoring of mechanically ventilated pediatric patients with sepsis, trauma, or major surgery. The study revealed significant differences between the methods used to predict EE. Thus, they have reported that the Schofield equation for EE cannot give a correct prediction in cases of critically ill patients, requiring further evaluation and monitoring methods for the metabolic status [47].

These results emphasize still more the importance of using electronic equipment for monitoring energy demand in these patients. The continuous monitoring of EE is based on the evaluation of VO_2_ and VCO_2_ using IC. An old system for monitoring the metabolic status of critically ill patients using IC is the Deltatrac II (Datex-Ohmeda, 2000, Helsinki, Finland). Unfortunately, this method is quite expensive and requires trained personnel to operate the equipment. A newer system is the gas exchange module for monitoring E-COVX, known as M-COVX (GE Healthcare/Datex-Ohmeda, Helsinki, Finland) [48–50]. McLellan et al. conducted a study on the metabolic monitoring of mechanically ventilated critically ill patients using M-COVX (Datex-Ohmeda, Helsinki, Finland). The study showed that M-COVX is highly accurate, increasing the interest in the integration of this module in the context of multimodal monitoring methods for critically ill patients [51].

Regarding the operating principle, E-COVX analyzes VO_2_ and VCO_2_ by the breath-by-breath method. Another important feature of this system is its high stability in monitoring mechanically ventilated patients with increased secretions or in the maneuvering and nursing of endotracheal intubation [10, 52, 53]. Briassoulis et al. conducted a study on the accuracy of the results obtained using E-COVX modules on mechanically ventilated critically ill patients with increased secretions. The study showed that the system reaches a steady state approximately 5 min after open endotracheal suctioning with high accuracy [54].

Kan et al. studied the effects of metabolic status on the outcome of critically ill patients. The patients included in the study were divided into three groups: underfed, who received less than 90% of the necessary nutrition; appropriate feeding, receiving nutrition with an error of plus or minus 10%; and overfed patients, who received more than 110% of the necessary. Following the study, a significant improvement in hemodynamic status was reported in patients whose nutrition was individualized and related to their needs, reducing both the time of mechanical ventilation and the time in the ICU [55].

A high percentage of critically ill polytrauma patients require prolonged mechanical ventilation. A number of studies have shown that providing the needed energy to a mechanically ventilated critically ill patient is directly related to shorter mechanical ventilation time and to a shorter time spent in the ICU. Askanazi et al. proved that malnourished patients have a more prolonged mechanical ventilation time because the respiratory epithelium does not recover as fast and because there is a significant delay in restoring the functionality of the respiratory muscles [56]. On the other hand, Dark et al. proved that in critically ill patients who are receiving excessive alimentation exceeding their energetic needs, the mechanical ventilation time is also longer. In this situation, the increased mechanical ventilation time was explained by the increase in VO_2_ and the increase in blood gas imbalances, such as imbalance of the PCO_2_/PO_2_ ratio [57]. A similar study was conducted by Bassili et al. and reported a significant increase in the time of mechanical ventilation in patients who did not receive optimal nutrition [58].

There are a few other EE-monitoring modules, but studies have reported lower accuracy of the EE values detected. Graf et al. conducted a study on various monitoring systems for EE. Deltatrac II (Datex-Ohmeda, Helsinki, Finland), CCM Express (Medgraphics), and Quark RMR (COSMED) were used in the study. They reported statistically significant differences in terms of result accuracy, with differences of up to 441 kcal in the detected EE values [52].

## Conclusions

The complex pathophysiologies associated with severe trauma lead to a significant decrease in the survival rate of critically ill trauma patients. The nutritional status of the patient is closely related to a series of specific pathophysiologies involved in the worsening of the clinical status and lowering of the survival rate of these patients. Monitoring of the metabolic status and optimizing the nutritional therapy according to the needs of each patient is necessary, as it contributes directly to shorter mechanical ventilation times, shorter ICU stays, lower costs, and lower mortality rates.
